# Using Cognitive Diagnostic Models to Evaluate the Two-Process Theory of Matrix Reasoning

**DOI:** 10.3390/jintelligence13020022

**Published:** 2025-02-15

**Authors:** Julian Preuß, Franzis Preckel

**Affiliations:** Department of Psychology, Trier University, D-54286 Trier, Germany; preckel@uni-trier.de

**Keywords:** figural matrices, rational item construction, construct validity, linear logistic test model, least square distance method

## Abstract

Figural matrices are widely used to measure reasoning ability. According to the two-process model of figural matrix reasoning, task performance relies on correspondence finding (linked to induction ability) and goal management (linked to working memory). Cognitive theory suggests that item characteristics (i.e., change rules and design principles of figural elements) are related to the two solution processes and impact item difficulties in a multiplicative, interactive manner. This study tested the multiplicative effect hypothesis by comparing two cognitive diagnostic models using additive and multiplicative effect estimations. A 26-item figural matrix test was administered to 633 high-ability individuals across paper-and-pencil and computer formats. The linear logistic test model (LLTM) and least square distance method (LSDM) were applied to Rasch and 2PL item parameters. Contrary to the multiplicative effect hypothesis, the additive LLTM model showed better item parameter reconstruction than the LSDM that includes multiplicative effects. These results suggest that change rules and design principles may independently contribute to the difficulty of figural matrices. Correspondence-finding demands may primarily arise from design principles, while change rules may primarily contribute to difficulty through goal management demands based on their number and complexity. The findings highlight the need to consider item components related to the phenomenological representation of figural elements when explaining solution processes of figural matrices. Implications for cognitive theory and item construction are discussed.

## 1. Using Cognitive Diagnostic Models to Evaluate the Two-Process Theory of Matrix Reasoning

Abstract reasoning refers to the capacity to understand complex concepts, identify patterns, and solve problems that do not rely on past learning experiences (Cattell 1963). Figural matrices are a well-established measure of abstract reasoning (e.g., Jensen 1998; Marshalek et al. 1983; for a critical reflection, see Gignac 2015) that have been incorporated in numerous tests, including the Wechsler Adult Intelligence (WAIS-IV; Wechsler 2008) and Stanford Binet Scales (SB5; Roid 2003). These items require test takers to analyze figural elements in a matrix, to infer their relationships, and to select the correct response to complete the matrix.

Carpenter et al. (1990) introduced a dual-process theory of solution processes in matrix reasoning. The first process, *correspondence finding*, is associated with induction ability. It involves inferring relationships between figural elements. The second process, *goal management*, is linked to working memory. It involves maintaining and organizing subgoals while solving the matrix. The dual-process theory implies that the two processes do not operate independently but rather build up on each other; thus, failure in either process prevents item solution. Item difficulty should therefore be the result of a multiplicative relationship between the two solution processes. Previous studies of the cognitive processes involved in solving figural matrices have relied primarily on correlations with external measures. They yielded mixed results, including inconsistent correlations with correspondence-finding abilities and both high and low correlations with working memory. However, correlations only offer limited information about the nature of solution processes, because they imply an additive relationship between solution processes and not a multiplicative relationship. That is, the multiplicative effect implied in Carpenter et al.’s (1990) theory has largely been neglected, and currently, there is no formal investigation of the multiplicative effect hypothesis available.

Rational item construction of figural matrices enables systematic variation in the demands placed on test takers’ correspondence finding (i.e., induction ability) and goal management (i.e., working memory) by manipulating specific item components, such as the number of rules according to which the figural elements change in a matrix. Cognitive diagnostic models (CDMs) offer a way to investigate how these item components contribute to an item’s psychometric characteristics and allow for the modelling of different relationships between item components, including additive and multiplicative effects. The linear logistic test model (LLTM; Fischer 1974, 1983) has been used to validate figural matrices. More recently, the least squares distance method (LSDM; Dimitrov 2007) has been proposed, which allows for the estimation of multiplicative effects of item components on psychometric characteristics. Unlike the additive estimation of the LLTM, the multiplicative approach of the LSDM better reflects the interdependence of solution processes posited by Carpenter et al.’s (1990) model. Despite its potential, the LSDM has not yet been applied to figural matrices. This study compares the appropriateness of the LLTM and LSDM for modeling rationally developed figural matrices to test this core assumption of Carpenter et al.’s (1990) model.

### 1.1. Figural Matrices and the Two-Process Theory of Matrix Reasoning

**Figure 1:**
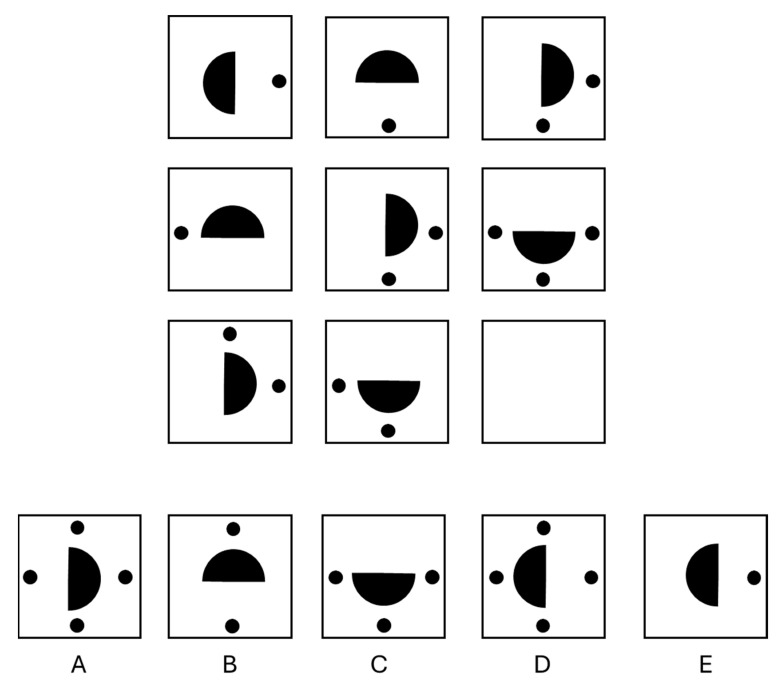
Illustrative example of a figural matrix item.

Figural matrices are a type of figural analogy where variations of figural elements are systematically arranged in a matrix according to predefined rules. Typically, the bottom right cell of the matrix is left blank, requiring test takers to identify the underlying rules and select the correct response from given alternatives. Figure 1 illustrates an example item with two rules. In this matrix, two types of figural elements are used: half-circles and dots. The half-circles rotate 90° both row-wise and column-wise from cell to cell (Rule 1), while the dots accumulate in the final cell of each row (Rule 2). Applying these rules, the empty cell should contain a left-facing vertical half-circle with four dots, one on each side, making response alternative D the correct answer.

According to the two-process theory of Carpenter et al. (1990), the first process, *correspondence finding*, involves inferring the transformation rule applied to the figural elements from one cell to another. For example, in the item depicted in Figure 1, the test taker must recognize that the dots sum up in the last cell while the half-circles rotate clockwise. Correspondence finding is an active exploration process, where similarities and differences between figural elements are identified through pairwise comparisons across cells. This process is associated with induction ability and is crucial for solving both single-rule and multi-rule items. The second process, *goal management*, encompasses the broader mechanism of sorting and managing subgoals and partial solutions when solving items with multiple rules. Items with more than one rule require the independent induction of each rule to form a comprehensive solution for the matrix. For example, in the item displayed in Figure 1, the test taker must understand that half-circles and dots follow different rules and articulate both rules to solve the matrix. Thus, the solution process is decomposed into subgoals, which are addressed sequentially. In multi-rule items, this leads to a stepwise process of inferring one rule after another while maintaining and integrating the subgoals of previous rules to arrive at a complete solution. This process is linked to working memory and is deemed particularly important for items involving multiple rules.

According to Carpenter et al. (1990), the two processes do not independently influence solution probabilities. In figural matrices involving multiple rules, induction and goal management occur sequentially, and failure in either process for any of the present change rules prevents the formulation of a complete solution. If induction fails and a rule cannot be inferred, the matrix becomes unsolvable regardless of successful management of subgoals and partial solutions. Conversely, even if all rules are inferred, failing to simultaneously represent all previously inferred rules also leads to an incomplete solution, rendering the matrix unsolvable. This implies that solution probabilities are the result of multiplicative rather than additive relations between solution processes. The probability of solving a figural matrix is not the sum but rather the product of all applicable change rules and design principles. Consequently, if any item feature remains unresolved, the entire item is unsolvable. This assumption is referred to as the *multiplicative effect hypothesis*.

### 1.2. Empirical Findings on Solution Processes in Figural Matrices

Carpenter et al. (1990) reported significant negative correlations between the number of rules in matrix items and their solution probabilities and high positive correlations between Raven’s Advanced Progressive Matrices (Raven 1976; RAPM) and tasks involving goal management. They concluded that while some induction ability is necessary for solving figural matrices, differences in working memory capacity might primarily determine performance on complex, multi-rule items (Carpenter et al. 1990, p. 415).

Studies investigating the relationship between working memory and matrix reasoning generally found significant positive correlations, but the strength of these correlations varies widely (Ackerman et al. 2002). Some studies report high correlations (e.g., Jastrzębski et al. 2018; Becker et al. 2016; Jarosz and Wiley 2012), suggesting that figural matrices largely measure working memory functions. Other studies found much lower correlations (e.g., Conway et al. 2002; Kane et al. 2005; Unsworth and Engle 2005), even lower than those typically seen with general intelligence measures (Ackerman et al. 2005).

Carpenter et al.’s (1990) original model emphasized the increasing demands on working memory with more complex, rule-heavy items. However, newer research shows that the relationship between working memory and performance on figural matrices does not consistently vary with the number of rules (Unsworth and Engle 2005; Wiley et al. 2011). Additionally, some studies indicate that working memory capacity does not consistently relate to item difficulty in figural matrices (Salthouse and Pink 2008; Unsworth and Engle 2005; Wiley et al. 2011). Researchers have suggested that learning efficiency for new rules during the test (Guthke and Stein 1996; Verguts and De Boeck 2002) and the ability to resolve interference and resist distractions when identifying novel rules (Conway 2005; Engle 2002; Wiley et al. 2011) play important roles. However, these accounts cannot explain the finding that pretest rule teaching tends to increase the relationship between figural matrices and working memory (Loesche et al. 2015). Teaching the rules in advance might reduce the variance explained by induction ability, thereby increasing the relative contribution of difficulties associated with working memory.

The findings regarding induction in matrix reasoning are even more ambiguous, and investigations into the role of induction abilities in figural matrices are scarce. Most studies have employed indirect methods, such as analyzing solution strategies. These studies generally confirmed that partial solutions are sequentially developed during task solution (e.g., Weber et al. 2023). Additionally, these studies generally indicate that most successful test takers use a systematic approach to formulate a holistic solution for the matrix (e.g., Arendasy and Sommer 2013; Becker et al. 2016; Pallentin et al. 2023). While these findings add plausibility to Carpenter et al.’s (1990) conceptualization of matrix reasoning and the role of induction ability, they cannot directly inform what abilities are associated with solution processes and how they mechanistically affect solution probabilities. Loesche et al. (2015) conducted one of few studies investigating correlations to abilities associated with correspondence finding. They found non-significant correlations between measures associated with induction ability and RAPM performance. Additionally, these relationships did not vary significantly when rule training was provided, though the association with working memory increased under this condition.

In summary, most studies have relied on correlations with other measures to elucidate the cognitive antecedents of figural matrices. While findings generally support a significant positive relationship between figural matrices and working memory, the nature of this relationship remains unclear, and the primacy of working memory in solving these items is not conclusively supported. Findings for induction ability suggest that this ability may not play a major role in solving multi-rule items, but there are only very few studies. Overall, the findings present an unclear picture regarding the relative importance of the two proposed solution processes in matrix reasoning. In addition, attempts to validate Carpenter et al.’s (1990) model by correlating figural matrix performance with abilities linked to the two solution processes overlook the central assumption of the model that the two processes do not independently influence solution probabilities.

### 1.3. Methodological Implications of the Multiplicative Effect Hypothesis

The multiplicative effect hypothesis has important methodological implications. If the relationship between solution processes and solution probabilities is multiplicative, correlations with overall task performance cannot reliably indicate how each process contributes to matrix reasoning because correlations assume linearity in relations, an assumption that is violated in multiplicative relationships. This limitation may help explain the inconsistent findings regarding the role of induction ability in matrix tasks. Despite the hypothesis’s importance for evaluating Carpenter et al.’s (1990) model and for interpreting validation studies, there has been no direct investigation of it, to the authors’ knowledge. Previous studies have largely relied on methods that assume an additive relationship between solution processes and solution probabilities. Drawing conclusions from such methods presupposes the validity of the additive model, rather than testing the multiplicative effect hypothesis or accounting for the possibility of non-linearity between item features and solution probabilities at the psychometric level. This creates a substantial gap in the current methodology used to understand the factors underlying matrix performance.

Addressing this gap requires investigating additive versus multiplicative effects at the item level by examining how item components related to the solution processes (i.e., change rules and design features of figural elements) affect solution probabilities. Cognitive diagnostic models grounded in rational item construction offer the necessary statistical framework, as they permit the examination of both additive and multiplicative effects of item components on solution probabilities. These models facilitate direct comparisons that can test the validity of the multiplicative effect hypothesis.

### 1.4. Rational Item Construction and Cognitive Diagnostic Models

Rational item construction is a systematic approach to creating test items based on cognitive theory. The process begins by defining a construct and explaining the solution processes on an item level. A taxonomy is then developed to describe all item components and their relation to the solution processes, guiding the construction of items. This rule-based approach predicts item characteristics based on construction parameters derived from the cognitive theory, ensuring content validity. The cognitive model and taxonomy used to construct items can be validated by employing cognitive diagnostic models (CDMs).

CDMs are an analytical framework where item responses are scaled using item response theory (IRT) and then deconstructed along the components of each item.^1^ In this CDM framework, it is assumed that if the cognitive theory correctly describes how item components affect solution processes, solution probabilities can be reconstructed using the underlying taxonomy of item components. This analytical framework opens multiple unique avenues of psychometric analysis. On the one hand, it allows one to validate a taxonomy for the rational construction of items with specific characteristics (e.g., very difficult or very easy items). On the other hand, this analytical framework also allows one to model how the item components related to working memory capacity or induction ability differentially affect solution probabilities. This can be used to analyze what processes are more relevant to item solution, but also how different solution processes jointly affect solution probabilities, making it a fitting tool for the validation of the multiplicative effect hypothesis.

A widely used cognitive diagnostic model (CDM) is the linear logistic test model (LLTM; Fischer 1983), which extends the Rasch model by incorporating item construction rules specified via a q-matrix. The q-matrix encodes, in integer form, the presence and prevalence of each component underlying an item, thus mapping the pre-established taxonomy onto the items. In the LLTM, item difficulties are modeled as additive linear combinations of component difficulty parameters, with weights given by the q-matrix entries. Accordingly, the *solution* probability P of an item j for a person i is given by

(1) $$P_{ij}=\frac{exp(\theta_{i}-\sum_{k=1}^{K}q_{jk}\eta_{k}-c)}{1+exp(\theta_{i}-\sum_{k=1}^{K}q_{jk}\eta_{k}-c)}$$

where $\theta_{i}$ denotes the person ability parameter and η represents the difficulty estimate of component k. The weights $q_{jk}$ indicate the extent to which component k is present in item j, and the normalization constant c ensures that the sum of item parameters is fixed at zero. This formulation mirrors the standard Rasch model while constraining item difficulties to align with the specified taxonomy. Consequently, the difficulty σ of item j can be expressed as the sum of all weighted item component difficulties (base parameters):

(2) $$\sigma_{j}=\sum_{k=1}^{K}q_{jk}\eta_{k}$$

The comprehensiveness of the taxonomy can then be assessed by the predictive accuracy of the theoretical q-matrix against empirical item difficulties, typically assessed through the correlation between item difficulty estimates from the LLTM and the basic Rasch model without weighted base parameters. A strong correlation would indicate a taxonomy’s effectiveness in capturing the determinants of item difficulty. Given the summative difficulty contribution of each item component, this model assumes an additive effect of each component and thus an additive relationship of the underlying solution processes. For example, when one component, such as a specific rule, cannot be identified within correspondence finding, the probability of solving the item would be expected to drop by a fixed effect defined by the difficulty estimation of this rule.

More recent research has developed non-parametrical CDMs that allow for the inclusion of non-linearity in the reconstruction process of item components, thereby accommodating Carpenter et al.’s (1990) multiplicative effect hypothesis and allowing for explicit comparison between reconstruction methods and effect assumptions. The least square distance method (LSDM; Dimitrov 2007) quantifies a components’ influence on item characteristics by minimizing the Euclidean distance between model-supplied and estimated item characteristics curves (ICCs). The LSDM is also based on the q-matrix. In contrast to the LLTM, the LSDM is supplied with item characteristic curves (ICCs) from an externally estimated IRT model (Rasch, 2PL or 3PL model). Depending on the IRT model, the LSDM then estimates item parameters of difficulty, discrimination, and guessing rate and reconstructs the submitted ICCs along the q-matrix so as to minimize the Euclidean distance between the supplied ICCs and the reconstructed ICCs across all items at fixed levels of the person parameter theta θ.

The LSDM thus assumes that the probability of answering correctly to an item is the *product* of the likelihoods of all item components, that is:

(3) $$P_{ij}=\prod_{k=1}^{K}{\left[P\left(A_{k}=1|\theta_{i}\right)\right]}^{q_{jk}}$$

where $P_{ij}$ is the probability of correct response on item j for a person at ability level $\theta_{i}$, $P\left(A_{k}=1|\theta_{i}\right)$ is the probability of correct performance in component $A_{k}$ for an examinee at the ability level $\theta_{i}$, and $q_{jk}$ is the weight of the q-matrix for item j and component $A_{k}$. $P_{ij}$ is estimated from a preexisting IRT model at pre-specified levels ($\theta_{i}$). Taking the natural logarithm on both sides of Equation (1),

(4) $$ln(P_{ij})=\sum_{k=1}^{K}q_{jk}ln(P\left(A_{k}=1|\theta_{i}\right))$$

given the IRT estimates of the item parameters, fixed theta levels, and n binary items, Equation (2) creates a system of n linear combinations with K unknowns at a given level of $\theta_{i}$. The matrix algebra form of this system of linear equations is

(5) L=QX

where L is the (known) vector with elements $ln(P_{ij})$, Q is the (known) q-matrix, and X is the (unknown) vector with elements $X_{k}=ln(P\left(A_{k}=1|\theta_{i}\right))$. The solutions for vector X are sought by minimizing the Euclidean norm of the vector $\left|\left|QX-K\right|\right|$ using the basic least square difference method from linear algebra. The fit of the model is then assessed using measurements of distance at the fitted points.

The LSDM supplements the LLTM in multiple ways, including the use of 2PL and 3PL ICCs, whilst the LLTM is limited to Rasch item difficulty estimations. The main difference, however, lies in the fact that the LSDM is based on a multiplicative effect estimation compared to the additive effect estimation of the LLTM. While the latter assumes that the probability of solving an item decreases linearly with the presence of each construction rule, based on its difficulty level, the multiplicative estimation of the LSDM suggests that the overall item solution probability becomes zero if any single component of the item has a zero probability of being solved. This estimation method aligns with Carpenter et al.’s (1990) model as it assumes that if correspondence finding fails and a rule cannot be identified, the item remains unsolvable, regardless of the difficulties of other rules or design principles.

Despite the overall utility of rational item construction and CDMs for understanding the cognitive antecedents of solution processes, a very limited number of studies have employed CDMs in the rational construction of figural matrices. Notable examples include Formann (1973), Hornke and Habon (1984a, 1984b, 1986), Embretson (1998), and Preckel (2003). More recent research has utilized CDM-like methodologies on figural matrix items in other contexts (e.g., Bartok and Kubinger 2023; Koch et al. 2022; Pallentin et al. 2023; Freund et al. 2008), particularly in the realms of automatic item generation. However, these studies have applied the LLTM or extensions of the LLTM. To the best of the authors’ knowledge, the LSDM has not yet been applied to a taxonomy for the rational construction of figural matrices, and therefore no formal comparison of the additive and multiplicative effect assumptions in the reconstruction of responses on figural matrices has been conducted.

### 1.5. A Taxonomy for the Construction of Difficult Figural Matrices

**Figure 2:**
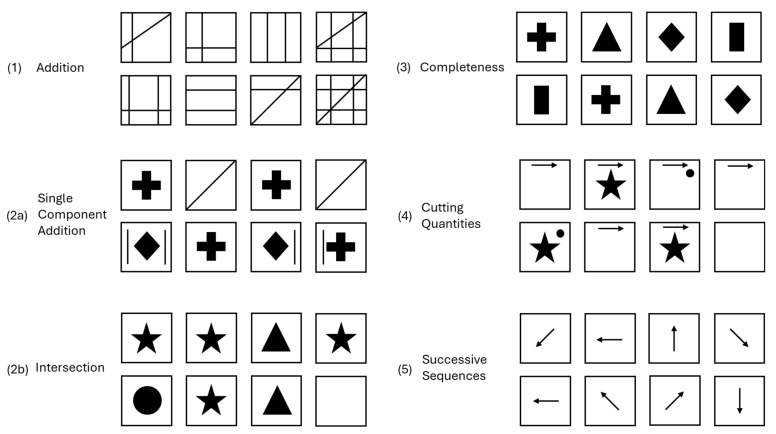
Examples of change rules for figural matrices (direction of rule application by row).

**Figure 3:**
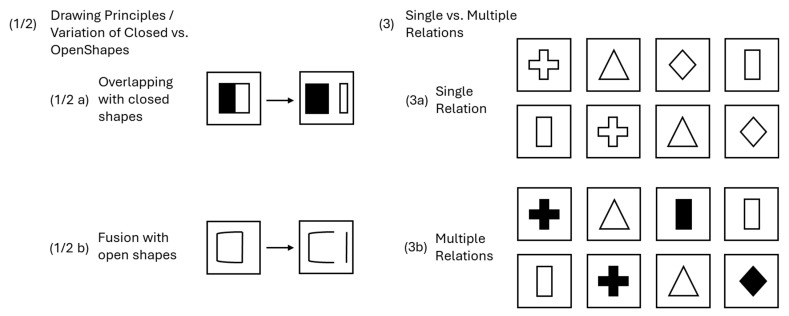
Examples of design principles for figural matrices.

Preckel (2003) conducted a comprehensive review of components for constructing figural matrices. Her goal was to identify rules suited to the construction of challenging items for the assessment of high-level reasoning abilities. Preckel’s (2003) selection was grounded in prior research on item difficulties, error rates, and process analyses, along with theoretical arguments based on Carpenter et al.’s (1990) model. Since the aim was to design especially difficult matrices, the selection was limited to rules applicable to 4 × 4 matrices. For the final taxonomy, she selected five change rules and four design principles for constructing difficult matrices in a 4 × 4 format. Examples for each change rule and design principle are depicted in Figure 2 and Figure 3, respectively.

#### 1.5.1. Change Rules

To illustrate the rules, we use a specific notation to represent figural elements and their modifications: letters (e.g., a, b, c, and d) denote different figural elements, while ‘Ø’ denotes an empty cell without elements, and ‘|’ separates different rows or columns within a matrix; single quotation marks behind letters refer to modifications done to the element.

(1) Addition (ADD): The figural elements affected by the addition rule sum up in the last cell in the direction of rule application. If the figural elements to be added are in identical positions, they are superimposed (ab - ac - ad - abcd).
(2) Addition with Null Element (ANE): ANE summarizes two rules that refer to conditional addition of elements in the last cell in the direction of rule application based on the distribution of these elements in prior cells. Since the conditions set by these rules are the exact opposite of each other, both rules are summarized under ANE.
  - Single Component Addition: In the direction of rule application, figural elements affected by the rule that occur only once are *taken over* in the last cell; elements that occur more than once are *hidden* in the last cell (a - b - a - b|abc - d - ac - db).
  - Intersection: In the direction of rule application, figural elements affected by the rule that occur only once are *hidden* in the last cell; elements that occur more than once are *carried over* to the last cell (a - a - b - a|b - a - c - Ø).
(3) Completeness (COM): In the direction of rule application, the cells contain different figural elements that appear exactly once and in a different order in other cells of a row or column (a - b - c - d|d - a - b - c).
(4) Cutting Quantities (CQ): In the direction of rule application, only elements present in all the previous cells are uniquely taken over to the last cell (a - ab - ac - a|bc - a - ab - Ø). CQ is only applicable to 4 × 4 matrices.
(5) Successive Sequences (SS): In the direction of rule application, various changes of figural elements (e.g., rotation) are performed continuously in the direction of rule application. Changes are either applied additively, meaning once for every cell, or may also be performed in ascending or descending numbers, meaning that the change is applied, for example, once from the first to the second cell, twice from the second to the third, etc. (a - a′ - a′″ - a″″″). For example, a figural element could be rotated 1 × 45° from cell one to cell two (a′), 2 × 45° from cell two to cell three, to a sum of three rotations (a′″), and finally rotated 3 × 45° from cell three to cell four, leading to a total of 6 × 45° rotations compared to cell one (a″″″).

#### 1.5.2. Design Principles

(1) Drawing Principles (DP): Systematic changes to the phenomenological presentation of figural elements are applied.
  - Overlapping: Two or more figural elements overlap.
  - Fusion: Two or more figural elements merge into one figure.
(2) Variation of Open Shapes (VOS): Variations are applied to open vs. closed shapes.
(3) Rule Correspondence/Single vs. Multiple Relations (MR):
  - Single relation: A figural element changes only according to one rule.
  - Multiple relations: A figural element changes due to multiple rules.
(4) Direction of Rule Application (DRA)
  - Row-wise: Rules of change are applied row-wise.
  - Column-wise: Rules of change are applied column-wise.
  - Row- and Column-wise: Rules are applied both row- and column-wise.

#### 1.5.3. Validation with the LLTM

Preckel (2003) constructed a figural matrix test based on her compiled taxonomy. A final test version of 26 matrices was submitted to a large sample of high-ability individuals between the ages of 13 and 58 to validate the taxonomy. A sample of individuals enrolled in schools or clubs for the intellectually gifted was chosen because the primary focus of the study was to validate a taxonomy for the assessment of very high reasoning ability. Eighteen Rasch-conform items were submitted to the LLTM. The correlation between the empirical item difficulties and item difficulties predicted by the LLTM was *r* = 0.91, meaning that 82.8% of variability in item difficulties could be explained by the underlying item components. These findings underscore the importance of a rule-based approach to item construction and the utility of the taxonomy in this context.

### 1.6. The Present Study

Carpenter et al.’s (1990) two-process model of matrix reasoning has mostly been explored using correlations to external criteria that cannot test a central assumption of the model: the multiplicative effect hypothesis. This study aimed to investigate the multiplicative effect hypothesis by comparing item response reconstruction from the LLTM and the LSDM applied to items that were rationally developed based on Preckel’s (2003) taxonomy.

First, response data on the rationally constructed figural matrices by Preckel (2003) were calibrated using IRT. The resulting item response characteristics were then reconstructed along the underlying taxonomy using the LLTM, based on additive effects of construction parameters, and the LSDM, using multiplicative effect estimations, representing the multiplicative effect hypothesis. The latter model was also applied to item characteristics derived from the 2PL model to explore the utility of incorporating item discrimination parameters into the reconstruction process.

Second, we assessed the appropriateness of the respective cognitive diagnostic models by comparing the degree of ICC reconstruction and the predictive validity for empirical item characteristics. Specifically, we evaluated whether the LSDM better explains item characteristics than the LLTM. If the multiplicative effect hypothesis holds true, the LSDM should yield superior item response reconstruction. Conversely, a superior fit of the LLTM would challenge Carpenter et al.’s assumptions.

Lastly, we inspected the size and rank order of difficulty parameter estimations for different change rules and design elements across the models, to support the interpretation of the previous model comparisons. These exploratory comparisons may provide additional nuances for interpreting differences between estimation methods in light of the underlying cognitive theory and offer guidance for future item construction.

## 2. Methods

### 2.1. Procedure and Participants

The study was based on the original data from Preckel (2003). The matrix items were submitted to 657 participants recruited from schools and clubs for the intellectually gifted in Germany. The items were administered in both paper-and-pencil (P&P) and online formats. In both presentation formats, participants were provided with an exercise booklet containing practice items and explanations of all item components; the test takers were encouraged to only begin with the test once all rules and design principles were understood. The reason for using the practice items was that the test takers should have comparable starting conditions in terms of knowledge of the item components when they start working on the actual items. On the one hand, this contributes to test fairness. On the other hand, the prior definition and communication of the item components establishes unambiguous solution spaces (Preckel 2003), which was expected to lead to more reliable and valid items (Arendasy and Sommer 2013; Embretson 1998; Verguts and De Boeck 2002).

The matrices were administered as a power test without a time limit. After excluding 24 datasets due to incomplete responses or non-serious engagement, data from 633 participants aged 13–58 (*M* = 20.15, *SD* = 8.28) were retained for analysis, with 429 datasets stemming from P&P and 228 from online assessments. The sample consisted of 430 males and 203 females. Test taking was voluntary. For the online test, *N* = 3004 persons initially clicked on the link distributed through mailing lists of organizations for the intellectually gifted; around 8% of them participated, indicating a strong self-selection effect in the online test. Individuals previously identified as intellectually gifted reported their previously assessed IQs (*n* = 110). In addition to those who reported their IQs from previous assessments, the RAPM (Raven and Court 1998) was administered to 64 students from schools for the gifted on a separate occasion following the assessment with the developed figure matrices. Reported IQs from previous assessments averaged 129.57 (*SD* = 13.69); IQs assessed with the RAPM averaged 125 (*SD* = 15.21).

### 2.2. Instrument

**Figure 4:**
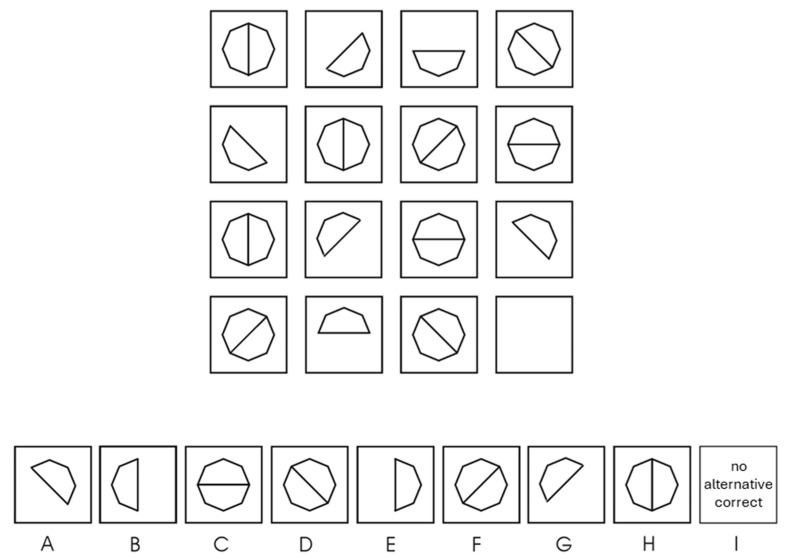
Example of a figural matrix item (taken from Preckel 2003). Note: In this example item, addition with null element is applied column-wise and successive sequences are applied row-wise to closed shapes using the drawing principle “overlapping”.

Preckel (2003) developed a matrix test based on the compiled taxonomy. In a first step, 189 figural matrices representing a full crossing of all two-rule combinations were constructed and submitted to a sample of university students. In the second step, the fourteen most difficult items were selected and extended by adding additional rules to the item stems. The final test comprised 26 figural matrices. All matrices were 4 × 4 with nine response alternatives: the correct solution, seven distractors systematically constructed following the approach by Vodegel Matzen (1994), and a ‘no alternative correct’ option to preclude falsification strategies (see Figure 4 for an example item). Item difficulties in the high-ability sample of the 657 participants recruited from schools and clubs for the intellectually gifted ranged from 0.27 to 0.84 (*M* = 0.59, *SD* = 0.17). The scale showed an internal consistency of McDonald’s ω = 0.85, as well as criterion validity of *r* = 0.53 for the APM (*n* = 64), *r* = 0.46 for reported IQs (*n* = 110), and *r* = 0.30 for math grades (*n* = 331). It should be noted that these correlations might be attenuated due to ceiling effects in the external criteria, as distributions in these values were left-skewed with multiple test takers scoring the scales’ maxima.

### 2.3. Data Analyses

All analyses were conducted using R (Version 4.2.2, R Core Team 2023), utilizing the eRm (Version 1.0.2, Mair et al. 2008) and sirt (Version 3.12-66, Robitzsch 2022) packages. The analysis code is available online at: https://osf.io/w29re/?view_only=c8907ad78e244c86aafb021ef3eb1095 (DOI: 10.17605/OSF.IO/W29RE) (accessed on 15 January 2025).

This analysis consists of multiple steps to prepare for the model comparison used to test the multiplicative effect hypothesis. First, items were calibrated using item response theory (IRT) to estimate each item’s characteristics. Second, both cognitive diagnosis models (CDMs) were used to reconstruct item responses based on the taxonomy used to create the figural matrices. Third, the appropriateness of each CDM was evaluated by examining how accurately the models reconstructed empirical item response characteristics. The models were then compared to determine whether the additive or multiplicative relationships among item components better explained the data, thereby informing the multiplicative effect hypothesis. Lastly, the difficulty estimates for the change rules and design principles were compared across models to facilitate interpretation of these effects. The four steps are described in more detail below.

In the first step (calibration), the raw item response data for the 26 figural matrices from the P&P dataset were scaled using the Rasch model. Item fit was assessed with Q-item statistics, and a subset of items conforming to Rasch model expectations was retained. Additionally, a 2PL model was fit to this same subset of items to ensure comparability between the Rasch and 2PL estimations. Both the Rasch and 2PL models were also fit to the same item subset in the online sample (used for later cross-validation).

In the second step (reconstruction), both CDMs were fit to the data or item characteristic curves (ICCs) derived during the calibration step. For the LLTM, the raw response data from the Rasch-conforming items were scaled again using the q-matrix of the item components (see Appendix A Table A1), resulting in reconstructed item characteristics based on the taxonomy. Because this approach relies on a Rasch-based methodology, item characteristics reflect only difficulty. For the LSDM, the ICCs obtained from the Rasch and 2PL models in the first step were reconstructed via the least-squares distance method, again using the same q-matrix. Thus, the reconstructed ICCs aligned with the taxonomy used to construct the figural matrices. Analyses related to reconstruction used only the P&P dataset, while the online data served for cross-validation.

In the third step, each model’s reconstruction was evaluated, and the models were compared based on their ability to reproduce empirical item responses. Three criteria assessed the quality of reconstruction: the Euclidean distance between the empirical and reconstructed ICCs, the correlation between empirically estimated and reconstructed item difficulties, and the predictive power of the reconstructed item characteristics when applied to item characteristics derived from the independent online dataset. The Euclidean distance was quantified by the mean absolute distance (MAD) between empirical and reconstructed ICCs at various focal points along the ability scale for each item, summarized as mean and median MAD for each model. Following Dimitrov (2007), a MAD below 0.05 was deemed indicative of good ICC reconstruction, and a MAD below 0.10 was deemed satisfactory. For the second criterion, the correlations between the empirically estimated and reconstructed item difficulties were calculated. In the LLTM, the reconstructed item difficulties were derived directly from the model, whereas for the LSDM, they were approximated via the fitted ICCs, using the ability value corresponding to a 0.50 solution probability. Finally, the model-based item difficulties were cross-validated by computing correlations with item difficulties in the independently sampled online dataset. To compare model adequacy, two types of significance tests were conducted. First, paired t-tests were applied to item-level MAD values. Second, correlations between model-implied and empirical item difficulties in both datasets were compared using Steiger’s Z-test (Steiger 1980), which accounts for interdependencies when comparing correlations.

As a robustness check, item difficulties were also predicted using a multiple-regression approach. Because the LLTM and LSDM rely on different reconstruction logics, any differences in results might partly reflect the estimation method rather than the effect assumption per se. To address this, a multiple regression was run in which the two most difficult change rules and design principles from the LLTM were used to predict Rasch and 2PL item difficulties. In a subsequent step, interaction terms between change rules and design principles were added to examine whether the interaction of item components contributed additional explained variance beyond their main effects. This approach follows previous research that has validated LLTM results through multiple regressions, including Preckel (2003).

In the fourth and final step, the item component parameters obtained from each model were compared. For the LLTM, the focus was on the “base parameter” capturing the additive shift in item difficulty. For the LSDM, component parameters, items, and ability levels were estimated on the same scale, allowing interpretation of how likely a component is to be solved at a specific ability level. Because the LLTM and LSDM use different scales, rank orders and their stability were analyzed to compare parameter estimates. Additionally, the LSDM-based component characteristic curves were visualized by transforming logit-solution probabilities into logistic functions. These curves, analogous to ICCs, depict how ability level relates to the probability of solving a particular item component. Their position and shape indicate each component’s contribution to item difficulty and discrimination.

## 3. Results

### 3.1. Calibration

Item parameters for the Rasch and 2PL model were aligned with the original findings by Preckel (2003) (see Appendix A Table A2 and Table A3). Item difficulties ranged from σ = −1.26 to σ = 1.99. Discrimination parameters ranged from β = 0.32 to β = 2.13 (*M* = 1.11). Difficulty estimates from the Rasch and 2PL calibration of the P&P data correlated *r* = 0.99, 95% CI [0.99, 1.00]. The 2PL model showed substantially better fit than the Rasch model as indicated by a significant likelihood ratio test and substantial improvements in relative model fit of χ^2^ = 141.75, *df* = 26, *p* < .001, ΔAIC = −89.75, ΔBIC = −173.44, ΔCAIC = −41.85 for the P&P and χ^2^ = 67.16, *df* = 25, *p* < .001, ΔAIC = −15.13, ΔBIC = −209.38, ΔCAIC = −97.11 for the online data. A total of 18 items showed fit based on Q-Item statistics under the Rasch Model. Some misfitting items were expected as the test was newly developed for the validation study of Preckel (2003). The subset of Rasch-conform items was used for all subsequent CDM analyses.

### 3.2. Reconstruction and Model Evaluation

When the LLTM was fit to the response data from the 18 Rasch-conform items, ICC reconstruction was excellent, with a mean MAD = 0.033 and median MAD = 0.024. At the item level, all items demonstrated at least sufficient ICC reconstruction with MAD < 0.10, and 16 of the 18 items exhibited very good to excellent ICC reconstruction based on Dimitrov’s (2007) guidelines. Item-level ICC reconstruction is detailed in Appendix A Table A4. Reconstructed item difficulties correlated *r* = 0.91, 95% CI [0.76, 0.96] with empirical item difficulties, indicating that 83% of the variability in item difficulties could be explained by the taxonomy using the LLTM with an additive effect estimation. For the cross-validation, LLTM difficulty estimates derived from the P&P data correlated *r* = 0.89, 95% CI [0.72, 0.96] with item difficulties from the independently sampled online data, underscoring the robustness of the model estimation and the predictive power of the taxonomy under this model.

The LSDM was also fit to Rasch ICCs from the same 18 items. ICC recovery was good, with a mean and median MAD of 0.059 and 0.054, respectively. The correlation between the fitted points of the reconstructed ICCs at .50 solution probabilities and empirical difficulties was *r* = 0.70, 95% CI [0.34, 0.88] for the P&P dataset and *r* = 0.73, 95% CI [0.40, 0.89] for the online dataset. This means that the taxonomy was able to explain 49% and 53% of variability in item difficulty by using a multiplicative effect estimation in the P&P and online data, respectively.

Item-level analyses revealed that only items 2 and 16 showed poor reconstruction, with MAD > 0.17. For an overview of MADs for each individual item, see Appendix A Table A4. After removing these items, ICC reconstruction improved to a mean and median MAD of 0.046 and 0.041, respectively, with no items showing insufficient reconstruction. The correlation between reconstructed and empirical item difficulties also increased to *r* = 0.85, 95% CI [0.61, 0.95] in the subset of fitting items. In the cross-validation, the correlation to empirical difficulties derived from the online data was *r* = 0.83, 95% CI [0.57, 0.94].

Although items 2 and 16 did not show statistically significant misfit under the Rasch model based on either Q-Item statistics nor the Euclidean distance to empirical ICCs, the LLTM analysis was replicated without these items to ensure comparability to the LSDM. In the subset of 16 items, ICC reconstruction for the LLTM improved to a mean and median MAD of .019 and .019. Predictive correlations for the LLTM also increased to *r* = 0.98, 95% CI [0.95, 0.99] for the P&P data and *r* = 0.96, 95% CI [0.87, 0.98] in the online data.

An LSDM model was further fit to the 2PL ICCs estimated from the 18 Rasch model conform items to explore the contribution of item discrimination in the reconstruction of response patterns. ICC reconstruction was “somewhat good” based on Dimitrov’s (2007) classification, with mean and median MADs of 0.073 and 0.066, respectively. Correlations to empirical item difficulties were *r* = 0.66, 95% CI [0.28, 0.86] for the P&P data and *r* = 0.60, 95% CI [0.18, 0.83] for the online data. This means that the taxonomy was able to explain 44% (P&P) and 36% (online) in empirical item difficulties under the multiplicative effect assumption when discrimination was also included in the model. Item-level ICC reconstruction is detailed in Appendix A Table A4. Most items showed good or very good reconstruction, with the exception of items 4, 14, 19, and 21, which exhibited “somewhat poor” or “poor” reconstruction with MAD > 0.10. Since there was no apparent relationship between these items, they were not excluded from the LSDM analysis.

### 3.3. Model Comparison

To evaluate the multiplicative effect hypothesis, the models with their respective additive and multiplicative effect assumptions were compared. The LLTM showed significantly better ICC reconstruction than the LSDM on Rasch data. This was indicated by a significantly lower Euclidean distance between reconstruction and empirical ICCs, with *t*_df = 17_ = −2.64, *p* = .02, *d* = −0.57, as well as significantly better prediction of empirical item difficulties with Z = 2.54, *p* = .011 and Z = 1.98, *p* = .048 following Steiger’s Z-Test for the P&P as well as online data, respectively.

Excluding the two items that could not be properly reconstructed under the Rasch-LSDM did not change this pattern. After removing these items, the reconstruction for both models improved. However, the improvement for the LLTM was larger than the improvement in the Rasch LSDM. In the reduced item set, the LLTM-reconstructed ICCs showed significantly lower item-level MAD than those reconstructed by the LSDM, with *t*_df=15_ = −4.14, *p* < .001, *d* = −0.97. The LLTM also predicted empirical item difficulties significantly better than the LSDM, with Z = 3.94, *p* < .001 and Z = 2.58, *p* < .001 for the correlations to item difficulties from P&P and online data, respectively. Thus, the LLTM using an additive reconstruction still showed superior ICC reconstruction compared to the multiplicative reconstruction method of the LSDM in the subset of items fitting the latter model.

In the third model, the LSDM was applied to ICCs from a 2PL, instead of a Rasch model. The inclusion of item-level discrimination led to a descriptively worse performance of the LSDM with regards to response reconstruction, but was not significantly worse than the Rasch LSDM (*t*_df=17_ = −0.79, *p* = .44, *d* = −0.30 for the MADs; *Z* = 0.72, *p* = .472 and *Z* = 0.52, *p* = .606 for the comparison between Rasch LSDM and 2PL LSDM predictions of item difficulties from P&P and online data, respectively).

Multiple regression analyses replicated this pattern of results. In neither model did the inclusion of interaction terms lead to a significant or otherwise noticeable increase in explained variance in item difficulty, indicating that the superiority of the additive effect estimation is not limited to the comparison between LLTM and LSDM. This was the case irrespective of the use of Rasch or 2PL difficulties. See Appendix A Table A5 for an overview of multiple regression analyses.

Taken together, the results indicate that the LLTM showed better reconstruction on all metrics than the LSDM. The LSDM also did not improve in reconstruction when ICCs from a 2PL model were used. This was independent from the specific item misfits and could be replicated outside the CDM logic using regression analysis. Refer to Table 1 for an overview of model comparisons.

### 3.4. Parameter Inspection

Mean-centered difficulty estimates for the item components across all three models, as well as mean-centered discrimination estimates for the 2PL LSDM model, are presented in Table 2. In the LLTM, drawing principles showed the largest contribution to item difficulty, followed by cutting quantities and successive sequences. Conversely, completeness, addition, and multiple relations contributed the least to item difficulty, with the remaining rules showing average difficulty contributions.

**Figure 5:**
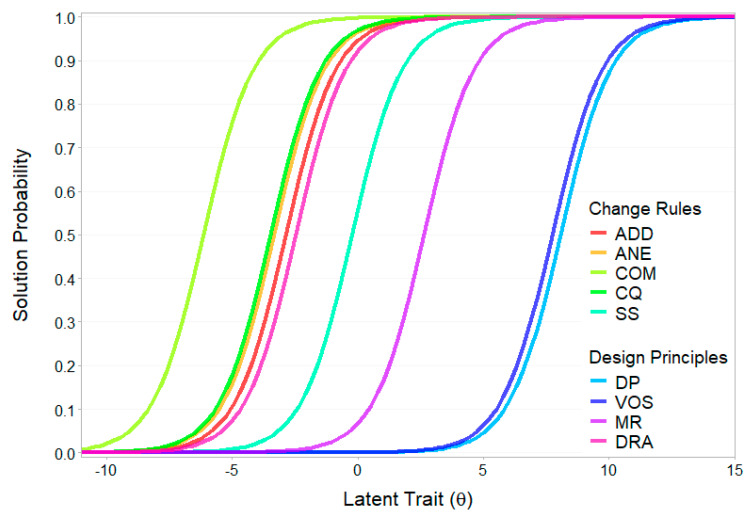
Logistic attribute characteristics curves for the nine item components from the LSDM applied to Rasch ICCs. Note: Logit solution probabilities of parameters fit to item ICCs were transformed to logistic functions to illustrate components’ impact on item characteristics. ADD refers to addition, ANE to addition with null element, COM to completeness, CQ to cutting quantities, SS to successive sequences, DP to drawing principles, VOS to variation of open shapes, MR to multiple relations, and DRA to direction of rule application.

Difficulty estimates from the Rasch-based LSDM model differed significantly from those of the LLTM. The rank order correlation between the LLTM base parameters and Rasch LSDM difficulty estimates for the item components was *r* = 0.55. In the Rasch LSDM model, drawing principles and variation of open shapes were by far the most difficult components, with estimates several standard deviations above the sample’s mean, followed by multiple relations. Cutting quantities showed average difficulty, while the remaining rules were estimated to be very easy, with difficulty estimates more than 2.8 standard deviations below the sample mean. Attribute characteristic curves for the item components based on this model are depicted in Figure 5. Notably, only drawing principles and completeness maintained the same rank order as in the LLTM, while successive sequences and multiple relations reversed their rank order positions.

Difficulty estimates for the item components derived from the LSDM applied to the 2PL ICCs differed significantly from the LLTM estimates and showed little overlap with the LSDM estimates derived from the Rasch model ICCs, with Spearman rank correlations of *r* = 0.54 and *r* = 0.01, respectively. In this model, variation of open shapes, multiple relations, and direction of rule application were the most difficult, with difficulty estimates between two and three standard deviations above the mean. Completeness, addition, and addition with null element showed average difficulties, while successive sequences, cutting quantities, and drawing principles were the easiest, with more than two standard deviations below the mean.

**Figure 6:**
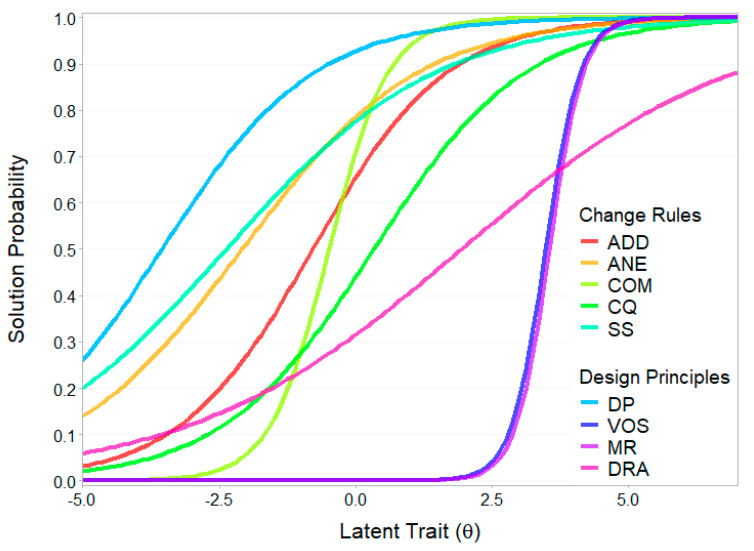
Logistic attribute characteristics curves for item components from the LSDM applied to 2PL ICCs. Note: Logit solution probabilities of components fit to item ICCs were transformed to logistic functions to illustrate components impact on item characteristics. A small jitter was introduced to MR and VOS to increase visibility, as these lines overlapped. ADD refers to addition, ANE to addition with null element, COM to completeness, CQ to cutting quantities, SS to successive sequences, DP to drawing principles, VOS to variation of open shapes, MR to multiple relations, and DRA to direction of rule application.

Regarding discrimination, most components showed moderate discrimination, with completeness, variation of open shapes, and multiple relations exhibiting high to very high discrimination, while direction of rule application showed poor discrimination. The very low rank correlations between the two LSDM models suggest no underlying systematic pattern in the change of rank orders. Component characteristic curves for the 2PL LSDM model are depicted in Figure 6.

## 4. Discussion

Figural matrices are an established and widely used measure of reasoning ability. Despite their popularity, the cognitive processes underlying these items remain partially unexplored. In particular, the interplay between the solution processes, the multiplicative effect hypothesis proposed by Carpenter et al. (1990), has not been sufficiently tested. In the present study, we addressed this gap by contrasting an additive model with a multiplicative model to determine which one better explains item responses on rationally constructed figural matrices.

To operationalize additive versus multiplicative assumptions, we compared two cognitive diagnostic models: the LLTM and the LSDM. The LLTM expresses item difficulty as the sum of independent difficulty contributions from each item component. The LSDM assumes that each component must be solved in order to solve the item, implying a multiplicative interaction. Contrary to the multiplicative effect hypothesis, we found that the additive LLTM yielded better item reconstruction and predictive accuracy than the LSDM, which performed comparatively worse in all criteria.

### 4.1. Additive vs. Multiplicative Effects

Our results support the additive effect model. Item difficulty appears to increase incrementally with each additional change rule or design principle. These findings may seem counterintuitive, as conceptually one might expect multiplicative effects (i.e., a near-zero probability of success if any rule is missed). There are several possible explanations for our findings.

First, methodological artefacts of the LSDM might have masked the superiority of the multiplicative estimation, causing the LLTM to emerge as superior instead. The LLTM and LSDM do not only differ in their effect assumption, but also in their reconstruction approach. Whereas the LLTM is an extended Rasch model, where difficulties are expressed as the sum of construction parameters, the LSDM uses a non-parametric procedure that minimizes the Euclidean distance between observed and reconstructed item characteristic curves across the ability continuum, imposing a product of probabilities for the underlying item components. These two estimation approaches differ in multiple ways. Specifically, the LSDM has no strict built-in structure, unlike the Rasch model, and may treat responses in a more free-form manner. Additionally, there is no maximum likelihood algorithm behind the LSDM with the constraints of a Rasch model. Instead, each item parameter is estimated independently from the others and only connected by shared item components. Therefore, item parameter estimation is not confined to any shared structure in the estimation process. Additionally, there is no penalty term in the least-square distance algorithm that would constrain model complexity or parameter values within the bounds of a pre-defined model as the Rasch model would. Moreover, since information of different items is not shared in the estimation, as it would be in a maximum likelihood approach, there is no pooling effect across items, which would minimize the impact of noise in individual response data. Instead, the LSDM simply applies the same minimization algorithm to all items at all focal points equally. Through all these mechanisms, the LSDM, with its increased flexibility, might be more prone to over-fitting and have more leeway to capture random fluctuations or idiosyncratic response patterns that might lead to suboptimal solutions regarding item response reconstruction on the scale level. Therefore, it could be argued that although a multiplicative effect might better describe the relationships between item components, the LSDM still underperforms compared to the LLTM, as it may be less able overall to account for variation within response patterns.

We know of no study that systematically addresses the robustness and optimal conditions for applying the LSDM. The strong discrepancy in parameter estimation between the LSDMs’ fit to Rasch and 2PL models of the same data seem to support the unstable nature of this estimation approach. However, the entirety of the results makes it seem unlikely that methodological artifacts alone account for the observed results. Specifically, removing misfitting items did not appreciably alter the findings, and a cross-validation with an independent online sample produced the same general outcomes. This was also true for the 2PL model. Furthermore, a simpler regression analysis showed no evidence of interaction effects as suggested by Carpenter et al. (1990). This lack of interaction, together with the other results hinting at the overall robustness of the LSDM estimation, indicates that the inferior performance of the LSDM likely reflects a broader empirical pattern in how item components interact within solution processes, rather than exclusively being an artifact of the LSDM itself.

Second, additional factors might affect solution processes so that test takers can achieve higher-than-chance accuracy even without inferring every single rule. Test takers might employ strategies to leverage other rules or distractors to increase their solution probabilities when induction fails. For instance, once a subset of rules has been inferred, test takers can narrow down the answer choices by focusing only on those answer options that incorporate these known rules, thereby increasing the likelihood of guessing correctly. Similarly, a verification-oriented approach can be replaced or complemented by a falsification strategy, in which an inferred rule is used to eliminate distractors that violate that rule. This process of testing each potential answer against the partially inferred rules can yield additional information about the likely correct solution by testing the rules of the distractors against the matrix. Heuristic techniques, such as identifying “near-match” patterns among the distractors, may further refine a guess by intuitively assessing how closely each candidate aligns with the partial solution. These strategies might explain how, even in the absence of a fully articulated matrix solution, the probability of success can exceed the near-zero level predicted by a strictly multiplicative relationship. Moreover, flaws in item construction may enable item solving with an incomplete matrix solution. If the correct response is set apart from the distractors by more than one rule, then identifying only some of those rules can be enough to choose the correct answer. Similarly, if distractors fail to incorporate certain rule components, test takers can infer the missing rules or solve the item by employing a falsification approach, rather than relying on a complete solution, as discussed with solution strategies. Moreover, multiple solution pathways may exist, allowing test takers to select the correct response without fully inducing all change rules. Both the availability of compensatory strategies and the characteristics of the distractors reduce the detrimental impact of failing to induce one or more rules. When distractors are leveraged through partial rule inference, solution probability no longer collapses to zero; instead, it aligns more closely with an additive effect.

While these accounts theoretically explain why solution probabilities need not collapse to zero when a given rule is not induced, they are unlikely to account for the present findings. In this study, distractors were also rationally constructed to contain a controlled number of partial solutions, and items were constructed to require a holistic understanding of the matrix, without redundant rules. The inclusion of a “no alternative correct” option further discouraged compensatory strategies, and all items underwent screening to eliminate multiple solution pathways. Under these conditions, partial solutions alone should not suffice to solve the items, rendering a reliance on meta-strategies improbable as an explanation for the observed results.

Lastly, because neither of these explanations convincingly accounts for why the LLTM outperforms the LSDM under an ostensibly suitable multiplicative framework, it appears that the multiplicative effect hypothesis does not hold in its preconceived form. Instead, the effects of item components on solution probabilities are more consistent with an additive pattern. This suggests that although some interaction between correspondence finding and goal management may occur in multi-rule items, it is not the primary source of difficulty. Rather, change rules seem to contribute mainly through their number, exerting a general difficulty effect. Design principles, in turn, add a separate main effect of difficulty that remains at least partially independent of the rules themselves. Thus, introducing a design principle to an item that includes a particular rule reduces the item’s solution probability by a relatively fixed amount. Any variation in this effect across rules effectively averages out on the scale, resulting in the stable additive pattern expressed by the LLTM. Consequently, the impact of design principles cannot be fully subsumed under change rules, as Carpenter et al. (1990) proposed. However, because the present study did not fully cross every change rule with every design principle—limiting the orthogonality of their estimation—conclusions about the interaction between these two categories of item components are confined to rejecting the multiplicative effect hypothesis in its current form and interpreting the overall pattern observed. Future research could employ experimental designs that systematically cross these item components within a CDM framework to further substantiate these findings.

### 4.2. Item Components: Change Rule and Design Principles

We found that design principles represented the most difficult item components, exhibiting considerably higher average difficulty than change rules. Although the rank ordering of item components showed limited consistency between the LLTM and the LSDM, design principles showed the highest difficulty contribution relative to other components in all models. Drawing principles remained the top contributor to item difficulty across models, and the gap between them and the other components widened substantially under the LSDM. In Carpenter et al.’s (1990) cognitive model, design principles and other variations of the phenomenological representation of figural elements were not included; only change rules were used in reconstructing responses to the RAPM in the original and many subsequent studies. The present results, however, indicate that design principles are critical determinants of item difficulty and must be accounted for when constructing and predicting item response patterns. The data further imply that design principles exert effects partly independent from those of change rules, consistent with the observation that these components respond differently to multiplicative effect estimation. This outcome supports both the general interpretation of the multiplicative effect hypothesis in this study and a broader body of research suggesting dissociable solution processes.

Various studies have also subsumed effects independent from goal management demands. Loesche et al. (2015) demonstrated that in items with design principles, item difficulty can in part be explained by the disruption of filtering functions of working memory. Specifically, selective encoding seems to be substantially hindered through design principles in figural matrices, constituting an individual pathway through which these construction parameters could impact item difficulties independent of goal management functions. This means that design principles, too, are associated with abilities that can be mechanistically separated from the demands of change rules.

In the past, multiple studies have supported the notion that performance on figural matrices may have multidimensional determinants (e.g., Dillon et al. 1981; Primi 2001; Snow et al. 1984; Vigneau and Bors 2005). Specifically, earlier studies have noted that figural matrices may be solved using more visuo-spatial processes versus using more verbal analytical processes. For example, Hunt (1974) proposed two distinct strategies for solving figural matrices based on their conceptual analysis of problem solving in these tasks: visual strategies, relying on mental imagery, as well as analytical strategies, relying on logic to compare operations, while solving figural matrices. Embretson (1998) developed a cognitive design approach to psychometric testing and found that visuospatial manipulations (e.g., rotation, transformation) and verbal-logical manipulations (e.g., pairwise comparisons) independently influenced performance. DeShon et al. (1995) have also shown that the use of visuo-spatial compared to verbal-analytic solution processes can not only be differentiated within test takers, but also within items. They not only indicated that both solution processes were relevant, but also that certain types of items are more susceptible to one process over the other. Although this and previous studies primarily focused on differences between rules rather than design principles, it is nonetheless plausible to extend this reasoning to design principles as well. This seems especially plausible since many factor-analytic studies of intelligence have previously indicated that figural matrices are also associated with visuo-spatial abilities (e.g., Carroll 1993; McGrew 2009; Schneider and McGrew 2018), which ultimately originate from characteristics of the phenomenological representation of figural elements, the systematic manipulations of which are subsumed under the category of design principles. This factor-analytic perspective, together with process-oriented studies, indicates that the antecedents of successful matrix reasoning might be multidimensional and that it is plausible that design principles could affect solution probabilities through pathways that are separable from change rules, which might be associated with different broad abilities.

Given the discussion of dissociating the pathways through which solution processes are differently affected by certain types of item components, one might ask how these processes can combine into a unidimensional scale, especially given that the multiplicative effect hypothesis was not supported. It is important to clarify that unidimensionality does not necessarily imply a single psychological process. Even within a unidimensional scale, multiple processes can operate in supplementary, compensatory, or cofactor roles. As Gittler (1999, p. 77) notes, “Whatever the individual combinations of primary abilities and co-factors may be, for the Rasch homogeneity of the test it is only relevant that the relative item difficulties (item parameters) are not differentially influenced”. Consequently, the fit of a unidimensional model only implies that solution processes are similar between individuals and does not, by itself, illuminate how processes interrelate at the item level; establishing these relationships requires item-level reconstruction, for example through extensions of the basic IRT model, as with the LLTM. It is therefore plausible that figural matrix reasoning is influenced by both induction and capacity-related abilities, depending on the item components, even within a unidimensional scale. Contrary to the original model, this perspective aligns with the broader literature indicating that the cognitive demands of figural matrices extend beyond working memory capacity.

### 4.3. Implications

Contrary to expectations, the results did not support the multiplicative effect hypothesis. Instead, an additive relationship best described how item components relate to solution probabilities. This outcome contradicts a central assumption of the most prevalent cognitive model for explaining figural matrix solution processes and carries implications for both theory and item construction.

Given the superiority of an additive reconstruction, it appears there could be an interactive process between correspondence finding and goal management, but that this interaction may not be the main source of item difficulty. Specifically, item components tied to both processes may, at least partly, affect item solution independently. Carpenter et al. (1990) acknowledged that in items containing many rules, induction demands might be minimal, and difficulty could be driven primarily by the strain on goal management (working memory) due to the number of rules (Carpenter et al. 1990, p. 415f.). This suggests a somewhat independent pathway from change rules to working memory demands, without necessarily invoking a pertinent induction component. The present findings indicate that the same principle may apply to design principles. There could be one or multiple ways in which design principles or characteristics of the phenomenological representations of figural elements contribute to item difficulty without interfering with the induction of individual rules, potentially through visuo-spatial processes or selective encoding demands. Notably, Carpenter et al. (1990) did not incorporate design principles in their original model. The present findings suggest that item difficulty cannot be accounted for solely by change rules and their correspondence-finding demands; design principles must also be recognized for their role in shaping item difficulty, and these tasks may be more nuanced and complex than previously assumed. Future research should investigate the specific mechanisms by which design principles affect solution processes, including those evoked by change rules, and adapt the cognitive model accordingly to deepen our understanding of how these items function as indicators of reasoning ability. Employing CDM methodologies in tandem with systematic manipulations of item features may offer promising ways to advance this line of inquiry.

A further implication is that design principles could tap processes that are at least partly separable from those influenced by change rules, which has relevance for previous inconsistent findings. It was originally speculated that inconsistencies in the literature might be attributed to the proposed multiplicative nature of the effect. The present findings did not support the hypothesis and instead endorsed an additive effect structure, which should in theory allow for valid interpretations of correlations with external measures. If, however, solution probabilities are not completely explained by change rules as indicated by Carpenter et al. (1990), but also independently influenced by design principles, then item components become an important moderator of associations with external measures. This aligns with research suggesting that figural matrices without design principles might engage processes different from those activated by matrices containing them. Differences in item construction could therefore help explain variable correlations with external measures or the absence of correlations with induction-related constructs. For instance, items lacking design principles may show stronger links to working memory capacity, whereas the inclusion of design principles might introduce another systematic source of variance that moderates this relationship. Future research should thus integrate both change rules and design principles when examining external correlations and preferably adopt a rational approach to item design to account for the effects of distinct construction parameters.

These findings also have bearing on item construction. If change rules and design features recruit partially separable processes, then the measurement construct—and thus its validity—can shift. Validity must always be evaluated relative to a theoretical framework describing the construct and its relationships to others. Since the discussion here extends the cognitive theory itself, pinpointing the most suitable construction approach remains challenging. Although mapping construction parameters onto visuo-spatial or verbal-analytic processes seems plausible, this remains speculative until further research validates such assumptions. It is therefore difficult to recommend specific designs for figural matrices at this stage. Nevertheless, the present study and previous work indicate that design principles reliably represent the largest contributor to item difficulty, making them particularly useful for constructing challenging items. Even if the processes are separable at a mechanistic level, they can still be measured on a single scale. Incorporating design principles is not expected to undermine a test’s overall validity, while higher demands through design principles would, at least theoretically, reinforce induction demands, an essential aspect for maintaining content validity in reasoning assessments, and potentially increase the involvement of visuo-spatial processes. More evidence is needed to clarify the precise underpinnings of each item component’s contribution to difficulty, but test developers should consider the value of design principles rather than relying solely on change rules.

Further research is necessary to examine the various explanations and implications proposed here. The present study suggests that the link between item components and item difficulty may extend beyond current conceptions of how rules interact during induction. Future investigations should seek to establish how design characteristics operate during problem solving and the cognitive abilities through which they influence solution probabilities. Employing rational item construction and CDMs, systematically varying item material across experimental conditions, and relating outcomes to external measures or neurocognitive data could be crucial in uncovering the mechanistic underpinnings of these tasks.

### 4.4. Limitations

This study represents the first attempt to test the multiplicative effect hypothesis proposed by Carpenter et al. (1990) at a psychometric level, leading to several important implications and future research directions. However, some limitations must be acknowledged.

First, the analysis was based on items from the test developed by Preckel (2003), according to her taxonomy. Consequently, any limitations inherent in the taxonomy and the developed test are carried over to the CDMs. Notably, the original test included a modest number of items relative to the complexity of the taxonomy. Although efforts were made to balance item parameters and minimize redundancies, fully crossing all parameters within a single test posed practical challenges. This limitation prevented orthogonal attribute parameter estimates and may have affected the robustness of the construction parameter estimates, particularly in the LSDM, which may be more prone to bias in its multiplicative effect estimation. Furthermore, not all items from the original test fit well during item calibration; only 18 of the 26 items were Rasch-conform, and therefore usable for the CDM comparison. This exclusion further reduced the number and representativeness of the items. If the misfitting items were excluded due to characteristics related to the solution processes, this may have narrowed the scope of the CDM analysis and potentially inflated predictive validities. Moreover, if the misfits were linked to construction parameters, their exclusion might have introduced bias, benefiting the LLTM (which is based on the Rasch model) while masking misfits that could have been explained by a more flexible estimation method. Although Preckel (2003) provided comprehensive explanations for most of the item misfits during the construction and validation process, some misfits remained unexplained, potentially creating a gap in the validation of the taxonomy. Despite these limitations, the matrix test developed by Preckel (2003) generally performed well, with sufficient reliability and validity, and appropriate fit of the corresponding IRT models, including the predictive validity of the taxonomy for item characteristics through the CDMs. However, a more comprehensive appraisal of the taxonomy will require replication in other assessments with a broader scope of items and construction parameters and further validation procedures.

Secondly, the taxonomy was designed with a focus on high ability levels, both in the selection of construction rules and the recruited sample. Only rules expected to contribute substantially to item difficulty based on prior research were selected for Preckel’s (2003) original taxonomy. This restricts the scope of the present study to a limited set of difficult rules. Consequently, the CDMs are limited to the effects of these rules and their potential interactions, which may differ when a broader range of rules is considered. Additionally, the difficulty estimations were derived from a gifted, self-selected sample. While it has been argued that solution processes for figural matrices do not significantly differ in gifted individuals (Preckel 2003), the specificity of the sample may affect the generalizability of the findings to broader populations and other assessments. Although the taxonomy was based on a comprehensive review of construction parameters, and the original validation findings aligned with existing literature from general populations, the findings of the present study should be interpreted with these idiosyncrasies in mind. Future studies should aim to replicate these findings with different item materials and samples.

Third, in the original study by Preckel (2003), participants were taught the change rules and design characteristics beforehand. Later studies used a rule-teaching approach to reduce induction demands in figural matrices and found that it increased the association with working memory, but did not alter correlations with induction ability (Loesche et al. 2015). Consequently, teaching the rules and design characteristics prior to testing may have shaped the relationship between item components and solution processes, contributing to the results reported in this study. The present results should therefore be interpreted with this methodological factor in mind, and future research needs to replicate the findings without this manipulation. Further work should also investigate the impact of rule teaching on the psychometric properties and solution processes of figural matrices.

Lastly, the comparison between estimation methods was based on comparisons between CDMs. To the best of the authors’ knowledge, this study is the first to apply the LSDM to figural matrices. While the LLTM is a parametric extension of the Rasch model that explains item difficulty through a combination of construction parameters, the LSDM nonparametrically reconstructs ICCs based on minimizing Euclidean distances using the same parameters. Although Dimitrov (2007) demonstrated the validity of this method, the LSDM is based on a completely different reconstruction logic than the LLTM, and it remains unclear to what extent the comparison between CDMs is affected by methodological idiosyncrasies of the respective models. In particular, it remains unclear whether the LSDM is more susceptible to noise or introduces specific biases due to its estimation method, or what methodological conditions are necessary to ensure comparability with parametric models. Consequently, some differences between the models might be attributable to inherent statistical differences between the methods. We have argued that methodological idiosyncrasies are unlikely to solely account for the present findings based on overall model fit, findings from the cross-validation, and our robustness check. Nevertheless, the existence of some systematic influences or biases cannot be completely excluded, and it is not entirely clear how well the LSDM can accurately represent the multiplicative effect hypothesis. Future studies should therefore seek to apply and replicate this method to a wider range of datasets, and work on extensions of parametric CDMs to include a variety of effect assumptions to further corroborate the findings of the present study.

## 5. Conclusions

The present study investigated the multiplicative effect hypothesis within the two-process model of matrix reasoning. The results did not support the hypothesis and instead revealed a more complex interplay among item components. Specifically, the data suggests that design principles and change rules can operate through relatively independent, additive pathways rather than simply interacting in a single multiplicative manner. This perspective aligns with broader research indicating that figural matrix reasoning involves at least two distinct processing routes. Although these findings do not definitively resolve how individual item components contribute to overall item difficulty, they point to promising avenues for future research. Accounting for manipulations of the phenomenological representations of figural elements appears essential to better explain how matrix items are solved and how their components connect to the presumed solution processes.

## Figures and Tables

**Table 1:** Comparison of model’s appropriateness for reconstructing empirical item responses.

Model	IRT-Model	MAD	Comparison Model	Difference (Cohen’s *d*)	Predictive *r*	Cross Validation *r*
1. LLTM	Rasch	0.033	-	-	0.91	0.89
2. LSDM	Rasch	0.059	1	−0.57 **	0.70	0.73
3. LSDM	2PL	0.073	2	−0.30	0.66	0.60

Note: MAD = mean absolute distance for the model. Significance and effect sizes refer to paired *t*-tests for item-level MADs. Predictive and cross-validation *r* refer to the correlation between reconstructed and empirical item difficulties from the same P&P data as well as independently sampled online data. ** *p* < .01.

**Table 2:** Parameter estimates for item components from the LLTM and LSDM.

Parameter	*BP_LLTM-Rasch_*	σ*_LSDM-Rasch_*	σ*_LSDM-2PL_*	β*_LSDM-2PL_*
Change Rules				
Addition	−0.366	−2.851	−0.781	0.816
Addition with Null Element	−0.218	−3.463	0.336	0.722
Completeness	−0.395	−6.132	−0.485	1.846
Cutting Quantities	0.407	−0.214	−2.360	0.524
Successive Sequence	0.187	−3.320	−2.079	0.623
Design Principles				
Drawing Principles	1.109	8.057	−3.359	0.718
Variation of Open Shapes	−0.135	7.730	3.485	3.176
Multiple Relations	−0.314	2.650	3.485	3.171
Direction of Rule Application	−0.101	−2.457	1.948	0.398

Note: BP_LLTM-Rasch_ = base parameter from the LLTM; σ = difficulty and β = discrimination parameters. All estimates were mean centered.

## Data Availability

Analysis code is available online at OSF (DOI: 10.17605/OSF.IO/W29RE): https://osf.io/w29re/?view_only=c8907ad78e244c86aafb021ef3eb1095 (accessed on 15 January 2025). Due to legal rights, data cannot be made publicly available. Data will be provided upon request.

## Note

1 We note that the term “cognitive diagnostic model” in more contemporary literature typically refers to confirmatory latent class frameworks (e.g., DINA, G-DINA), while earlier literature referred to any model decomposing performance in terms of underlying cognitive operations as CDMs. Authors who develop and use these extended IRT models to deconstruct item response patterns on the psychometric level still use the original terminology of CDMs (e.g., Dimitrov 2007; Dimitrov and Atanasov 2012). Therefore, the latter definition was adopted for the present study.

## Appendix A

**Table A1:** q-Matrix for the 18 Rasch-conform items.

**Operation**	**1**	**2***	**3***	**4***	**5***	**6***	**7***	**8**	**9***	**10**	**11***	**12**	**13**	**14***	**15***	**16***
Rule																
ADD	0	0	1	0	1	0	1	1	0	0	0	1	1	1	1	0
ANE	1	1	1	1	0	1	1	1	1	2	0	2	0	0	0	1
COM	0	0	1	1	1	0	1	0	1	0	1	0	1	3	0	1
CQ	0	1	0	0	0	1	0	0	1	1	0	1	1	0	1	1
SS	1	0	0	0	1	1	1	0	0	1	1	0	0	0	0	1
Design																
DP	1	1	0	1	0	0	1	0	0	0	0	0	0	0	1	1
VOS	0	0	1	1	0	1	1	0	1	0	0	0	1	1	0	0
MR	0	0	0	0	1	0	1	0	0	0	0	0	0	1	0	1
DRA	1	1	0	1	1	0	1	0	1	1	0	1	1	0	1	1
**Operation**	**17**		**18***		**19***		**20**		**21***	**22***		**23**	**24***		**25***	**26***
Rule																
ADD	2		0		0		1		1	1		0	1		1	1
ANE	1		1		1		0		0	1		2	0		1	1
COM	1		0		0		1		2	1		0	1		0	1
CQ	0		1		1		1		0	1		1	0		1	0
SS	1		0		1		0		0	0		1	2		1	1
Design																
DP	0		0		1		0		0	0		0	0		0	1
VOS	1		0		1		1		0	1		1	0		1	1
MR	1		0		0		1		1	0		0	1		0	1
DRA	1		1		1		1		0	1		1	1		1	1

Note: ADD refers to addition, ANE to addition with null element, COM to completeness, CQ to cutting quantities, SS to successive sequences, DP to drawing principles, VOS to variation of open shapes, MR to multiple relations, and DRA to direction of rule application. Rasch-conform rules used for analysis are marked with an asterisk.

**Table A2:** Item parameters for the Rasch (1PL) and 2PL model of the paper-and-pencil data.

	1PL		2PL		
Item	σ	χ^2^-*p*	σ	β	χ^2^-*p*
1	1.23	0.02	1.09	0.61	.39
2	1.36	.72	1.45	1.27	.79
3	0.19	.35	0.21	1.30	.27
4	1.02	.16	0.98	0.91	.22
5	1.99	.75	1.83	0.74	.94
6	−0.34	.56	−0.32	0.83	.90
7	1.74	.02	1.57	0.66	.32
8	0.00	.01	−0.01	0.32	.64
9	−0.33	.04	−0.32	0.98	.05
10	1.93	.07	1.78	0.75	.43
11	−0.46	.13	−0.47	1.14	.11
12	0.67	.14	0.83	1.70	.24
13	0.08	.01	0.12	2.13	.11
14	1.89	.03	1.89	1.06	.03
15	−1.21	.10	−1.26	1.2	.41
16	−0.62	.01	−0.59	0.92	.01
17	1.10	.06	1.38	1.77	.36
18	−0.97	.02	−0.92	0.89	.05
19	−0.46	.25	−0.44	0.96	.31
20	0.64	.13	0.75	1.53	.53
21	1.82	.07	1.99	1.36	.06
22	−0.02	.81	−0.02	1.25	.80
23	0.36	.01	0.34	0.79	.10
24	1.03	.79	1.10	1.26	.80
25	0.10	.21	0.11	1.44	.63
26	1.25	.47	1.24	1.01	.51

Note. σ = IRT difficulty parameter, β = IRT discrimination parameter; χ^2^-*p* = *p*-value of the χ^2^- statistics of the Q-Item test.

**Table A3:** Item parameters for the Rasch (1PL) and 2PL model of the online data.

	1PL		2PL		
Item	σ	χ^2^-*p*	σ	β	χ^2^-*p*
1	2.14	.46	2.33	1.42	.56
2	1.88	.70	1.76	0.88	.88
3	0.64	.82	0.59	0.78	.95
4	1.38	.01	1.39	1.13	.01
5	2.87	.47	2.73	0.91	.53
6	−0.33	.00	−0.30	0.85	.02
7	2.74	.42	3.00	1.45	.24
8	0.41	.00	0.34	0.30	.82
9	−0.11	.05	−0.10	1.22	.02
10	2.18	.66	2.28	1.27	.61
11	0.06	.39	0.07	0.85	.78
12	1.69	.25	2.01	1.71	.38
13	0.77	.02	1.23	2.69	.08
14	2.74	.66	2.92	1.36	.68
15	−0.79	.70	−0.78	1.09	.67
16	0.06	.11	0.07	1.01	.17
17	2.18	.88	2.67	1.82	.99
18	0.11	.26	0.12	0.96	.53
19	0.14	.33	0.15	1.30	.37
20	1.41	.12	1.36	0.96	.04
21	2.87	.06	3.12	1.41	.07
22	0.85	.40	1.00	1.64	.90
23	0.77	.07	0.78	1.12	.09
24	1.80	.49	1.90	1.29	.51
25	1.29	.12	1.50	1.62	.09
26	1.92	.02	1.72	0.69	.15

Note. σ = IRT difficulty parameter, β = IRT discrimination parameter; χ^2^-*p* = *p*-value of the χ^2^-statistics of the Q-Item test.

**Table A4:** Item-level reconstruction measured by mean absolute distance for the LLTM and LSDM.

Item	LLTM	Rasch-LSDM	2PL-LSDM
2	0.097	0.201	0.081
3	0.022	0.029	0.041
4	0.027	0.067	0.168
5	0.049	0.069	0.065
6	0.055	0.077	0.030
7	0.006	0.024	0.099
9	0.01	0.061	0.011
11	0.001	0.031	0.067
14	0.043	0.006	0.132
15	0.054	0.068	0.056
16	0.037	0.166	0.019
18	0.016	0.026	0.058
19	0.061	0.066	0.114
21	0.015	0.037	0.138
22	0.014	0.078	0.046
24	0.021	0.027	0.038
25	0.054	0.013	0.070
26	0.014	0.022	0.084

**Table A5:** Multiple regressions of item components on item difficulties from Rasch and 2PL models.

Dataset	Model/Predictors	Unstandardized Coefficients	β	*p*	R^2^
Rasch	Main Effect				
	Intercept	1.126		.014	
	CQ	−0.945	−0.454	.019	
	SS	−0.212	−0.123	.481	
	DP	−1.193	−0.573	.007	
	VO	0.324	0.156	.356	0.663
	Intercept	1.220		.111	
	CQ	−1.001	−0.485	.592	
	SS	−0.300	−0.174	.168	
	DP	−1.233	−0.592	.793	
	VO	0.238	0.115	.941	
	CQ*DP	−0.066	−0.032	.868	
	CQ*VO	0.160	0.078	.852	
	SS*DP	0.150	0.087	.996	
	SS*VO	−0.004	−0.002	.286	0.666
2PL	Main Effect				
	Intercept	1.526		.003	
	CQ	−1.068	−0.488	.012	
	SS	−0.141	−0.077	.648	
	DP	−1.178	−0.538	.008	
	VO	0.445	0.203	.225	0.675
	ME + Interaction				
	Intercept	1.588		.051	
	CQ	−1.215	−0.556	.215	
	SS	−0.211	−0.116	.711	
	DP	−1.282	−0.586	.161	
	VO	0.423	0.293	.649	
	CQ*DP	0.297	−0.136	.746	
	CQ*VO	−0.176	0.080	.859	
	SS*DP	−0.011	0.006	.996	
	SS*VO	0.270	−0.123	.778	0.683

Note: Dummy codings for the presence of each item component were regressed on Rasch and 2PL item difficulties of the 18 Rasch-conform items used for CDM analysis. The inclusion of interaction effects did not increase explained variance in either dataset. Differences were non-significant with *F*(4, 9) = 0.02, *p* = .999 and *F*(4, 9) = 0.06, *p* = .993 for Rasch and 2PL difficulties, respectively. CQ refers to cutting quantities, SS to successive sequences, DP to drawing principles, VOS to variation of open shapes. Interaction terms are denoted with an asterisk between variables.
